# Cognitive LF-Ant: A Novel Protocol for Healthcare Wireless Sensor Networks

**DOI:** 10.3390/s120810463

**Published:** 2012-08-02

**Authors:** Marcelo Sousa, Waslon Lopes, Francisco Madeiro, Marcelo Alencar

**Affiliations:** 1 Institute for Advanced Studies in Communications (Iecom), Federal University of Campina Grande (UFCG), Campina Grande 58429-900, Brazil; E-Mails: waslon@dee.ufcg.edu.br (W.L.); malencar@dee.ufcg.edu.br (M.A.); 2 Polytechnic School of Pernambuco, University of Pernambuco (UPE), Recife 50720-001, Brazil; E-Mail: madeiro@poli.br

**Keywords:** wireless sensor networks, ant colony optimization, fuzzy inference, opportunistic access, healthcare networks

## Abstract

In this paper, the authors present the Cognitive LF-Ant protocol for emergency reporting in healthcare wireless sensor networks. The protocol is inspired by the natural behaviour of ants and a cognitive component provides the capabilities to dynamically allocate resources, in accordance with the emergency degree of each patient. The intra-cluster emergency reporting is inspired by the different capabilities of leg-manipulated ants. The inter-cluster reporting is aided by the cooperative modulation diversity with spectrum sensing, which can detect new emergency reporting requests and forward them. Simulations results show the decrease of average delay time as the probability of opportunistic access increases, which privileges the emergency reporting related to the patients with higher priority of resources' usage. Furthermore, the packet loss rate is decreased by the use of cooperative modulation diversity with spectrum sensing.

## 1. Introduction

Sensor devices can be embedded in a variety of medical instruments for use at hospitals, homes, and supply patients and their healthcare providers information about physiological and physical health states that are critical to the detection, diagnosis, treatment, and management of ailments. Much of modern medicine would simply not be possible without sensors such as thermometers, blood pressure monitors, glucose monitors, and imaging sensors [1]. In emergency response scenarios, for instance, a challenge is the design of a medical sensor that can deliver suitable functionality (e.g., sensor data transmission rate, type of data transmitted) to meet the evolving patient, provider and workflow needs [2].

Wireless sensor networks consist of low-power devices that integrate limited computation, sensing, and radio communication capabilities [3]. This technology may present impact on many aspects of emergency medical care [4]. Sensor devices can be used to capture real-time vital signs from a large number of patients, relaying the data to handheld computers carried by emergency medical technicians, physicians, and nurses. Body Sensor Networks (BSN) can be designed for prophylactic and follow-up monitoring of patients during hospitalization or in emergencies [5]. In a mass casualty event, sensor networks can greatly improve the ability of first responders to select and treat multiple patients equipped with wearable wireless monitors [6].

A large number of proposals currently available for telemedicine are based on wireless networks working in the license-free spectrum. One common problem observed by networks working in this part of spectrum is the increasing difficulty of quality of service (QoS) provisioning [7]. Currently, the license-free spectrum has been crowded by several networks. Transmissions in the license free bands can be affected by interference from other networks sharing the same spectrum, making it very difficult to predict the quality of service, posing a major problem for a health monitoring system which should support traffic with strict QoS requirements [8].

Dynamic spectrum access stands as a spectrum-efficient communication approach for wireless sensor networks due to their event-driven communication nature, which generally yields bursty traffic that depends on the event characteristics. In addition, opportunistic spectrum access may also help the deployment of multiple overlaid sensor networks and eliminate collision and excessive contention delay incurred by dense node deployment. Incorporating cognitive radio capability in sensor networks yields a new sensor networking paradigm, *i.e.*, cognitive radio sensor networks (CRSN) [9].

Cognitive technologies and their implementations can be inspired by the intelligent and well-organized behaviour of biological group of social insects [10,11]. In [12], the authors proposed Linguistic Fuzzy Ant (LF-Ant), a cross-layer [13] bio-inspired protocol which optimizes the election of cluster-heads [14] in wireless sensor networks. LF-Ant uses the fuzzy heuristic information, which deals with the imprecisions in distance measurements, by the employment of a fuzzy inference system. The adapting of LF-Ant operation to a CRSN designed to healthcare monitoring should take into account a cognitive component for detecting the spectrum opportunities.

The bio-inspired systems can still be extended for routing [15] algorithms' design and for the formation of hierarchical structures in medium access control (MAC) protocols. In [16], the authors performed tests to prove the pedometer hypothesis, which states that desert ants use a kind of step counter (an odometer mechanism) to optimize the travelling between food and nest. It is possible to draw a parallel with a hierarchical structure in forwarding and reporting the health status and those experiments accomplished over leg manipulations.

Apart from upper network layers' design, the fading caused by multipath in wireless channels affects the quality of transmission and increases the Packet Loss Rate (PLR). However, the reliable transfer of data in medical monitoring systems has crucial importance. Therefore, error resilient network schemes for medical data transmission should be developed for increasing network reliability [17]. Cooperative modulation diversity (CMD) can help in mitigating the fading effects without incurring waste of bandwidth or energy. CMD rotates the angle of the signal constellation, and interleaves the transmitted component symbols [18,19]. Between the interleaving intervals, the spectrum sensing may be used to detect the presence of higher priority users.

In this paper, the authors present techniques to optimize the performance of healthcare sensor networks, by proposing new approaches of biological inspirations. The main contributions of the paper are:

- The design of a novel clustering protocol, the Cognitive LF-Ant, which extends the operation of LF-Ant scheme, by enabling cognitive capabilities. Inspired by the behaviour of the ants *Iridomyrmex humilis* [20], the protocol uses the ant colony optimization to guide the clustering process.
- A novel protocol for intra-cluster emergency reporting based on the results of experiments performed with the ants *Cataglyphis fortis* [21]. The manipulation of legs in those experiments inspires the design of a hierarchical structure of medium access control, to attribute priorities in reporting the emergency status and the measured data, in accordance with each emergency degree (ED).
- An inter-cluster cooperative modulation diversity scheme with spectrum sensing. The goal is to decrease the packet loss rate, increasing the quality of health status transmissions, in parallel with spectrum sensing to assure the detection of higher priority users.

The remaining of the paper is organized as follows: some related work is commented in Section 2; the main concepts related to healthcare CRSN and to the system assumptions are discussed in Section 3; the proposed clustering protocol, Cognitive LF-Ant, is introduced in Section 4; the intra-cluster emergency reporting algorithm is presented in Section 5; the inter-cluster communication algorithm is presented in Section 6, in which the cooperative modulation diversity scheme with spectrum sensing is explained; in Section 7, the authors show the performance evaluation of the proposed scheme and the paper is concluded in Section 8.

## 2. Related Work

Monitoring the medical status of the people is the most widely studied application type of pervasive healthcare systems. The commonly used vital signs are electrocardiography, pulse oximetry, body temperature, heart rate, and blood pressure. Most of the studies focuses on capturing and sending the data to a remote site for further evaluation [17].

Malan *et al.* [6] introduced the CodeBlue system, a wireless infrastructure intended for deployment in emergency medical care, integrating low-power, wireless vital sign sensors, PDAs, and PC-class systems. CodeBlue enhances first responders' ability to assess patients on scene, ensure seamless transfer of data among caregivers, and facilitate efficient allocation of hospital resources.

Chen *et al.* [22] presented a concept for the second-generation Radio Frequency Identification (RFID) system and qualitatively demonstrated the value of its application in healthcare systems. They discussed benefits that the proposed 2G-RFID-Sys can provide, including improvements in system scalability, information availability, automated monitoring and processing of sensitive information, and access control.

In [5], the authors present an architecture for context-aware service discovery in a healthcare scenario that exploits synergy among intelligent agent technology, semantic web models and computational intelligence techniques. The architecture emphasizes the need of synergic approach between ontology and fuzzy logic to model the user's context.

Healthcare monitoring applications require emergency event reporting besides periodic physiological data reporting. Under emergency conditions, the data should be guaranteed to be delivered with a reasonable delay. For this purpose, data prioritization mechanisms should be developed. Moreover, the fairness among different emergent situations should be considered. Benhaddou *et al.* [23] propose a MAC scheme for healthcare which incorporates a preemptive service scheduling algorithm into the IEEE 802.11e QoS MAC to provide the highest and preemptive channel access precedence for medical emergency traffic. The authors describe the general architecture and propose a MAC scheme for healthcare sensor networks (MACH) that adapts channel provisioning based on the information criticality. However, the QoS-aware MAC protocols for low power wireless sensor networks like IEEE 802.15.4 must be considered in a healthcare specific perspective [17].

In [8], the authors study the performance of supporting telemedicine traffic in a CRSN. They introduce an infrastructure-based CRSN for telemedicine, in which cognitive base stations sense available spectrum and forward data for associated healthcare stations. They also present strategies and performance for supporting urgent and real-time periodic telemonitoring traffic in the network. The results show that satisfactory real-time performance can be achieved for telemedicine traffic. However, the authors do not consider the impact of the proposed system in other QoS parameters, like packet loss rate. Moreover, the infrastructure-based operation may not be feasible in emergency networks, because they may require *ad-hoc* network capabilities, like self-organization and distributed operation.

A powerful trend in the development of emergency healthcare systems is the employment of cognitive technologies which can be based on opportunistic spectrum access, as in [8]. Another cognitive perspective is based on intelligent systems and bio-inspired heuristics. A recent approach that represents the latter cognitive class is the LF-Ant protocol, which combines the organized behaviour of ants with the modelling by a fuzzy inference system [12]. LF-Ant is based on the operation of AntNet, a classical Ant Colony Optimization (ACO) algorithm related to a generic routing problem in communication networks [24,25].

Since the proposed protocol is based on LF-Ant, a brief description of the AntNet operation is necessary. In AntNet, each ant searches for a minimum cost path between a pair of nodes, *i* and *d* [24,25]. If an ant *κ* is in node *i*, it hops to *j*, in accordance with a decision rule that is a function of the ant's memory, ℳ*^κ^*, and of the local ant-routing table, 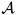
*_i_*. That table is obtained by a composition of the pheromone trails, *τ_ijd_*, and of the heuristic information, *η_ijd_*. Once the ant *κ* has completed a path, it deposits an amount of pheromone, Δ*τ^κ^*, proportional to the goodness of the path it built. In this way, after reaching its destination node, the ant moves back to its source node, along the same path, and increases the pheromone intensity, modelled by

(1) $$\tau_{\text{ijd}}\leftarrow \tau_{\text{ijd}}+\Delta \tau^{\kappa }$$

In order to prevent a premature convergence to non-optimal solutions, the pheromone of outgoing connections evaporates, indicated by

(2) $$\tau_{\text{ijd}}\leftarrow \frac{\tau_{\text{ijd}}}{\left(1+\Delta \tau^{\kappa }\right)}\forall j\in N_{i}$$

in which 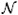
*_i_* is the set of node *i* neighbours. The relation between 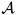
*_i_*, and the ant's decision rule, $p_{\text{ijd}}^{\kappa }$, is given by

(3) $$p_{\text{ijd}}^{\kappa }\{\begin{array}{ll} A_{i}=\frac{\omega \tau_{\text{ijd}}+(1-\omega )\eta_{\text{ijd}}}{\omega +(1-\omega )(\left|N_{i}\right|-1)} & \text{if}\;j\notin {ℳ}^{\kappa }\\ 0 & \text{if}\;j\in {ℳ}^{\kappa } \end{array}$$

in which *w* ∈ [0, 1] is a weighting factor between τ*_ijd_* and *η_iijd_*, and the denominator is a normalization term. The ant's memory, ℳ*^κ^*, indicates the set of nodes visited by the ant, and its use can avoid the occurrence of loops.

## 3. Healthcare Cognitive Wireless Sensor Networks

Sensor networks provide the technology to bridge the gap between patient load and available resources. When faced with a large number of casualties, the goal is to first care for those patients who will benefit the most from trauma care and fast surgical intervention. To accomplish this, emergency medical technicians can deploy wireless, low-power vital sign sensors to monitor severely injured patients. Using sensors, triage in the field and triage at the hospital can be made interactive by continuously feeding patient capacity information from a sensor network to a decision support mechanism. Such an infrastructure would allow efficient matching of out-of-hospital caseload to critical, hospital-based trauma facilities and resources. For example, field personnel could be alerted to locate more quickly those victims who were initially physiologically stable, but who subsequently deteriorate. The same sensor network would track stable patients from triage, through treatment, to their final hospital destination, all the while conveying vital sign and location data to the host system [6].

Emergency response scenarios, such as disasters, present several challenges to sensors due to unique patient, user, and environmental needs. The number of patients can increase to a large value, and high usage demands for wireless data exchange can easily overload existing radio communication channels. When a disaster occurs, the chaotic setting of limited resources, unreliable communication infrastructure, and inadequate information produces an organizational problem for care provider teams and prevents them from providing quality trauma care. These complex conditions require highly adaptable sensor solutions that can be efficiently deployed to the evolving user and workflow requirements, with minimal need for manual configuration [2].

Building a cognitive radio network can be a promising approach to provide emergency reporting services with strict QoS requirements. By dynamically changing its operating parameters, cognitive radio senses the spectrum, determines the vacant frequency bands, and makes use of these available frequency bands in an opportunistic manner, improving the overall spectrum utilization. With these capabilities, cognitive radios can operate in licensed (over a primary network) as well as in unlicensed bands (over a secondary network, also denoted cognitive network). In licensed bands, wireless users, with a specific license to communicate over the allocated band (the primary users), have the priority to access the channel. Cognitive radio users, denoted secondary users, can access the channel as long as they do not cause interference to primary users [9].

The low utilization of the licensed spectrum leaves a large amount of resource that can be used for cognitive radio networks. By opportunistically utilizing a wide range of spectrum unused by the primary networks, a sufficient available bandwidth can offer telemedicine traffic with high bandwidth and low latency requirements. The MAC protocol and resource allocations can be designed based on the specific requirements of the services and network conditions in order to satisfy the various QoS requirements for telemedicine services, while efficiently utilizing the radio resources. In addition, using a cognitive radio network reduces the cost of accessing the licensed spectrum [8].

### 3.1. System Assumptions

The cognitive healthcare network considered in this paper is composed by cognitive radio sensor nodes coupled with the patients' bodies, which measure vital signs and can identify their emergency degrees. A medical body area network can provide small area connection issues around human body, especially for the medical applications [26]. Many types of sensors can be employed to generate an alarm in accordance with the health status of each patient. In the proposed system, the Emergency Degree (ED) is split into three classes: Low ED, in which the patient does not present a risk of death and its health status can be considered regular; High ED, in which the patient has alterations in their vital signs, and presents moderate risk of death; and Critical ED, in which the patient presents an expressive risk of death because its health status is critically irregular. The method used to generate an ED diagnosis based on the values measured by the sensors is not taken into account in this paper. Techniques to generate ED data can be found in [1,8,17].

All the patients are considered as network nodes and they are deployed randomly over a specific region. Therefore, several nodes have to report the correspondent EDs and the measured vital signs information to a central station (sink node), which can be connected to hospitals, clinics, or research institutes over Internet links. In this paper, only the reporting from the sensor nodes to the sink node is considered. The nodes compose clusters and elect a cluster-head (CH) and a vice cluster-head (Vice-CH) for each cluster. The clustering process is based on the Cognitive LF-Ant algorithm.

The cluster-head aggregates the measured information (and its ED) from several nodes, which are inside the cluster. The Vice-CH helps the cluster in the reporting of High ED and Critical ED cases, over primary bands. The intra-cluster emergency reporting is considered free of transmission errors.

The Vice-CH can still perform beaconing functions to aid the cluster-head activities:

1. Inform the cluster-head about an emergency reporting;
2. Report the spectrum opportunities (e.g., carrier frequency, bandwidth, error rate), if the cluster-head requires;
3. Synchronize data transmissions.

## 4. Cognitive LF-Ant

LF-Ant is a clustering protocol designed for wireless sensor networks, which is based on the behaviour of ants that need to find optimal paths from the source to a final destination. The main goal is the optimal election of cluster-heads, in each round, and control the possible further cooperative retransmissions. The protocol modifies the classical modelling of the ACO algorithm, AntNet, since it translates the representation of vertices into edges. Therefore, each sensor node, vertex, in the network that uses the LF-Ant protocol can be seen as a path (edge), in the AntNet system. Then, the election of the best sensor node as a cluster-head, by the cluster, is equivalent to a choice of the best path from a source to a final destination, by an ant [12].

In this paper, the authors propose the Cognitive LF-Ant protocol, which extends the capabilities of LF-Ant, by inserting the cognitive operation required for adapting to a network with dynamic spectrum usage. That operation may provide quality of service and may optimize the reporting of health status from the monitored patients to a medical central station.

Consider a graph 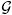 (*V, E*), in which *V* is the set of sensor nodes in a cluster that are candidates to become a cluster-head. The equivalent in the AntNet system is the set of candidate edges (paths) between two vertices, *i.e.*, the nest and the food source. In Cognitive LF-Ant, *E* represents the set of communication links (edges). That set has two possibilities: the primary (licensed) link and the secondary (cognitive) link. In AntNet, the ants go from the nest to the food source and backward several times, finding new paths, according to the environment changes. In Cognitive LF-Ant, the links, and which nodes use them, are chosen according to wireless channel variations and to the occurrence of an emergency status of a patient.

The operation of the proposed scheme starts with the random deployment of artificial ants in the monitored region. Each cluster receives an ant, *κ*, that indicates the respective first elected cluster-head. In the next elections, each cluster node runs decision rules and generates special values, denoted by chance. The clustering scheme based on chance variable is presented in Algorithm 1. For each sensor node, the respective ant, *κ*, travels to the final destination and moves back to the nest increasing the pheromone intensity, *τ_s_*. In the sensor network domain, this is equivalent to each sensor node running Expression (1). The update variable, Δ*τ^κ^*, indicates the quality of the chosen path by the ant. This can measure how good was the operation performance of the sensor node in the previous round. In the proposed protocol, the update variable is

(4) $$\Delta \tau^{\kappa }=\frac{\varepsilon_{s}\cdot \rho_{s}}{\zeta_{s}\cdot \Gamma_{s}}+\frac{\alpha_{s}}{N}$$

in which *ε_s_* is the residual energy of the node, *ζ_s_* is the energy consumed by the node in the previous round, *Γ_s_* is the total number of transmissions completed by the node in the previous round and *ρ_s_* = 2, if the previous transmitted packet was successfully recovered by the next destination node. Otherwise, if even with retransmissions the previous packet was not correctly recovered, *ρ_s_* = 1. Those values were determined empirically [27]. The total number of spectrum holes detected until the previous round is given by *α*, and *N* represents the total number of rounds up to that current time, in which the spectrum sensing has been needed.

The update variable presented in Expression (4) deals with components related to energy consumption, packet loss behaviour and with a cognitive component, which is the greatest difference between the LF-Ant and the Cognitive LF-Ant approaches. It privileges the nodes that can identify more spectrum opportunities at the primary side. The differences between outputs of spectrum sensing performed by the cognitive sensor nodes may be attributed to shadowing, hidden terminal problem and nodes' failure.


**Algorithm 1:** The Cluster-head (CH) and Vice-CH election.
**for**
*s* ← *1*
**to**
*#nodes*
**do** *τ_s_ ← τ_s_*_+_ Δ*τ^κ^*; 
$\tau_{s}\leftarrow \frac{\tau_{s}}{\left(1+\Delta \tau^{\kappa }\right)};$ Determine the value of the linguistic variable, 
eta_*s*_; *η_s_* ← defuzzification of 
eta_*s*_; 
$p_{s}^{\kappa }=\{\begin{array}{ll} A_{s}=\frac{w\tau_{s}+(1-w)\eta_{s}}{w+(1-w)(\left|N_{s}\right|-1)}, & \text{if}\;s\notin {ℳ}^{\kappa },\\ 0, & \text{if}\;s\in {ℳ}^{\kappa }, \end{array}$; 
${\text{chance}}_{s}\leftarrow p_{s}^{\kappa }$; Advertise the value of 
chance_*s*_; Receive the other advertisements and compare them; **if**
*the value of 
chance_s_ is greater than of the others*
**then**  Advertise a 
CH message; **else**  **if**
*the value of 
chance_s_ is the next higher value*
**then**   Advertise a 
vice CH message;  **else**   Select the closest CH as its coordinator;   Join the cluster.  **end** **end****end**


After the trail update, all the sensor nodes run Expression (2), which corresponds to the pheromone trails evaporation. The next step is the processing of the heuristic information, *η_s_*, given by the output of a fuzzy inference system, explained in the next section. By combining *τ_s_* and *η_s_*, the sensor node computes the value of the decision rule, according to Expression (3). This value indicates the probability that a path attracts ants and, equivalently, indicates the probability that a sensor node elects itself as a cluster-head.

Each node states the decision rule value as the variable chance*_s_*. Then, the nodes advertise a message to the other candidates, with that value attached, and wait messages from other nodes. If its value is higher than the chance from other nodes, the sensor node advertises a CH message, which means that the sensor node elected itself a cluster-head. If a node which is not a cluster-head receives a CH message, it selects the closest cluster-head as its coordinator and sends a message to join that cluster.

The sensor node which has the next higher value of chance (the second place in the election) becomes the vice cluster-head. That node reaches a relaying threshold, and the only chance greater in that cluster is the chance of the elected cluster-head (the first place in the election).

### 4.1. Fuzzy Heuristic Information

In ACO algorithms, the heuristic information represents a local information which does not depend on the quality of previous iterations. It represents a priori information about the problem instance or run-time information provided by a source different from the ants. In many cases the heuristic information is the estimate of cost (or its inverse) of adding the component or connection to the solution under construction [28].

In Cognitive LF-Ant, the heuristic information *η_s_*, represented by the variable eta, relates two other variables: local_distance and CH_dispersion [12].

The sum of distances between the candidate node and other nodes within a specific radius of transmission is represented by the variable local_distance. The greater the sum, the higher the energy to transmit the sensed data to the candidate node. On the other hand, the variable CH_dispersion is the sum of distances between the candidate node and the cluster-heads within a radius of transmission. However, the greater the sum, the higher the chance to coordinate the cluster. Besides promoting a good distribution of cluster-heads, it balances the transmission loads and the network processing. The estimation of positions and distances is done by the received signal strength intensity (RSSI), at a set-up phase.

Since eta is the combination of two variables based on imprecise measurements of distance, the fuzzy logic is well suited to represent its final processing [29]. Fuzzy logic is a mathematical tool that relies on qualitative variables, in contrast to the quantitative nature of crisp values [30]. In WSNs, besides the uncertainty of distance measurements by RSSI, the positioning of the sensor nodes can be subjected to little changes, which can be compensated in the fuzzy inference operation [27].

**Figure 1.**
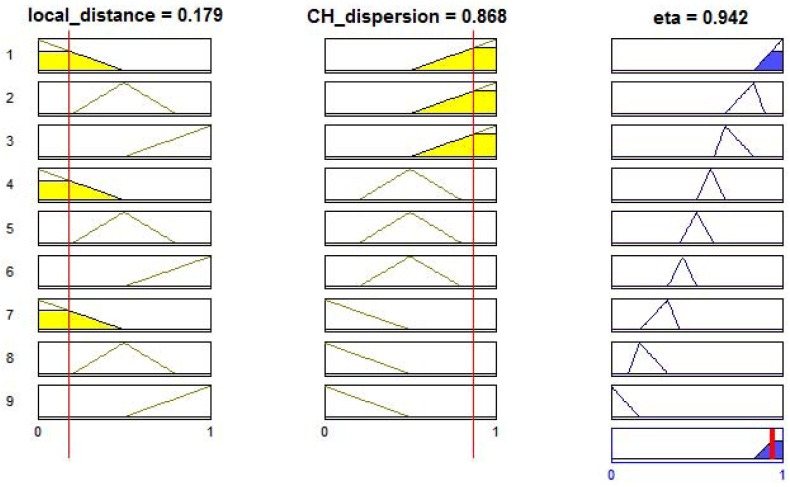
The eta defuzzification after computing the input variables, local_distance and CH_dispersion, over nine fuzzy rules.

Fuzzy logic decisions are based on IF-THEN rules, which are used to determine the value of output variables using approximate reasoning [31]. Furthermore, a fuzzy neural network can be used to acquire fuzzy rules based on the learning ability of neural networks and solve the lack of adaptability for possible changes in the reasoning environment [32]. The inference system is composed by *linguistic* variables and logical operators. The fuzzy rules used in the proposed protocol, to generate the value of the heuristic information, are presented in Table 1. Once the values of local_distance and CH_dispersion become small (Close) and great (Far), respectively, the fuzzy heuristic information presents the higher values, and thus, the greater chances to elect a cluster-head. The fuzzy variable eta is defuzzified (transformed to a crisp number) by the use of the Center of Area (CoA) method, which corresponds to calculating the centroid of the fuzzy output [33]. The membership functions adopted in this work are formed by triangles due the requirement of processing simplicity. The interaction between the fuzzy rules, over the values of local_distance = 0.179 (Close) and CH_dispersion = 0.868 (Far), is illustrated in Figure 1. The defuzzified value of 0.942 is equivalent to a Very High value of eta and to the point which splits in two equal parts the output area.

## 5. The Emergency Reporting

The foraging Saharan desert ants, *Cataglyphis fortis*, use a mode of dead reckoning known as path integration to monitor their current position relative to the nest. This enables them to return by a direct route, rather than retracing the tortuous outbound journey performed when searching for food items in their desert habitat [16].

In healthcare networks, the reporting of sensed information can be performed over different ways, using different transmission parameters, in accordance with the emergency degree of a patient. The proposed protocol attempts to design a hierarchical structure of dynamic resources' usage, that is based on experiments for testing the hypothesis that navigating ants measure distances travelled.

According to the pedometer hypothesis, ants that have travelled to the feeder on normal legs and have had their leg length modified at the feeder should cover a different distance on their homebound journey. Those experiments were accomplished by the manipulation of the legs' lengths and, hence, the stride lengths, in freely walking ants. Animals with elongated (“stilts”) or shortened legs (“stumps”) presented larger or shorter strides, respectively, and concomitantly misgauge travel distance. Travel distance was overestimated by experimental animals walking on stilts and underestimated by animals walking on stumps. Moreover, the experimental results indicated that, after the inclusion of a compensation due to the methods employed, the ants speed was proportional to the leg length [16].

In this paper, those relations between the ants' speed and their legs' length inspire the design of the proposed hierarchical structure of dynamic resource usage. As in desert ants, different users should reach different performance results in healthcare networks. More specifically, the MAC protocol and resource allocations should be designed specifically for telemedicine traffic in order to both effectively support the QoS requirements of the traffic and efficiently utilize the channel resources. The proposed emergency reporting scheme relates the resource allocation to the emergency degree of the patients. This is presented in Algorithm 2 and each case is detailed as follows.


**Algorithm 2:** The emergency reporting performed inside a cluster.
**for**
*n* ← 1 **to**
*#non–elected*
**do** Check the emergency degree (ED); **if**
*(ED_n_* == *critical) or (ED_n_* == *high)*
**then**  perform spectrum sensing;  **if**
*a free band (FB) is available*
**then**   % share the medium in the primary network;   send an 
emergency_message over FB;   wait for the detection of the CH or of the Vice-CH;   **if**
*ED_n_* == *critical*
**then**    send the measured data over the maximum packet size;   **else**    send the measured data over the restricted packet size;   **end**  **else**   % share the medium in the secondary network;   Access the secondary network over a CSMA protocol;  **end** **else**  % share the medium in the secondary network;  Access the secondary network over a CSMA protocol; **end****end**

- **Low ED—**The reporting of measured data by a patient with Low ED can be viewed as the walking of ant with stumps, which, in the network domain, could be equivalent to the use of the secondary network, only, which presents a high probability of collisions and poor quality of service. In this situation, the transmission has the lowest priority, if compared with the reporting of higher ED cases. The lower the speed of the ant with stump, the lower the priority in reporting the data;
- **High ED—**The reporting of patient's data with High ED presents a higher priority and tends to use more network resources than in the previous case. It is equivalent to experiments using ants without manipulation in legs (normal legs), which present bigger strides and are faster than stumps' ones. In this situation, there is an intermediate priority of spectrum access, *i.e.*, patients with High ED have more rights in use network resources than those with Low ED, but have less rights than those with Critical ED. They can access primary spectrum opportunities to transmit data, but the block transmission size (in Bytes) are, in most cases, shorter than in the cases with Critical ED. Consequently, in the transmissions performed by nodes with High ED, there are more spectrum sensing slots, which enable the detection of patients with Critical ED, or of licensed users. Actually, the representation of how less precedence a High ED node presents before a Critical ED node is given by
  (5) $$\text{BSR}=\frac{{\text{BS}}_{\text{H}}}{{\text{BS}}_{\text{C}}}$$
  in which BSR is the block size ratio for the opportunistic primary spectrum usage, BS_H_ is the block size for data transmission allowed to a High ED node (over an opportunistic access), and BS_c_ is the block size for data transmission allowed to a Critical ED node (over an opportunistic access). A small value of BSR indicates that High ED nodes have, continuously, less access to the primary spectrum, in favour of Critical ED nodes. For instance, if BSR is equal to zero, then it means that High ED nodes have no permission to opportunistically use the primary spectrum. In this case, all the spectrum opportunities detected are allocated to Critical ED nodes. On the other hand, if BSR is equal to one, then both High ED and Critical ED nodes have the same right to data transmission over the primary bands.
- **Critical ED—**The ants with stilts presented bigger strides in the odometer testing. After the compensation of the effect caused by the added load of glue and pig bristles on their legs, ants with stilts are characterized by the highest performed speed, and the reporting of patient data with Critical ED might be represented by the move of those ants. Those nodes have the highest priority (in the healthcare network) and should use more transmission resources. As for High ED nodes, Critical ED nodes are significantly helped by the respective CH and Vice-CH, because they can sense the emergency requests over the primary bands, by the spectrum sensing. The clustering formation based on the Cognitive LF-Ant is decisive for the election of the nodes that can efficiently detect more primary spectrum opportunities.
- **Primary Users—**They have absolute priority of spectrum access and must not be damaged by any cognitive user.

**Algorithm 3:** Communication between the clusters. The cooperative modulation diversity with spectrum sensing is invoked if errors occur.
**for**
*i* ← 1 **to**
*#packets*
**do** CH transmits a new packet; Receiver checks the CRC; **if**
*CRC*== *correct*
**then**  Receiver sends back an ACK; **else**  Receiver sends back a NACK;  *N_r_* = 0;  **for**
*j* ← 1 **to**
$N_{r}^{\text{max}}$
**do**   CH transmits *Q*_2_ and *I*_1_ in the time slot 1;   Vice-CH performs spectrum sensing in the time slot 1;   Vice-CH transmits *Q*_1_ and *I*_2_ in the time slot 2;   CH performs spectrum sensing in the time slot 2;   **if**
*A request to emergency reporting is detected*
**then**    Vice-CH forwards the emergency information;    CH transmits the rest of the packet by simply QPSK;   **else**    Both CH and Vice-CH keeps cooperating;   **end**   Receiver checks the CRC;   **if**
*CRC* == *correct*
**then**    Receiver sends back an ACK;   **else**    Receiver sends back a NACK;   **end**  **end** **end****end**


The inter-cluster routing is also inspired by the behaviour of foraging Saharan desert ants. The protocol is based on the criterion of shortest path, between the cognitive sensor node and the sink node. The information is forwarded from one cluster to another, towards the sink node. Then, the routing strategy uses the information of distance between the clusters, to determine the shortest path to the sink node. That relaying process can be viewed as the employment of the odometer mechanism on a setup phase, in which the distances of possible routes could be measured. In practice, the nodes may compare the distances and choose the shortest one. Furthermore, the forwarding direction must be kept towards the sink node, as if a navigation apparatus could be accessed. The union of those two characteristics avoids the occurrence of path loops and optimizes the choice of routes from the source cluster to the sink node. The proposed inter-cluster routing mixes a node state based stage, which is represented by the election of the nodes (performed by the Cognitive LF-Ant), with an oriented (towards the sink node) distance based routing (performed by the “Odometer mechanism of ants”) over those elected nodes in clustering formation.

The inter-cluster routing guides the information fused by the cluster-head. The Vice-CH may cooperate if errors occur, but both should detect new emergency reporting requests and they should be able to forward the data associated with them. That process is presented in Algorithm 3, whose details regarding digital modulation aspects are presented in the next section.

## 6. Cooperative Modulation Diversity with Spectrum Sensing

Concerning the biological behaviour of ants, a group transmission can be seen as an efficient way to carry a large prey to the nest, since ants working as a group can carry close to ten times the carrying capacity of a solitary ant [34].

In the proposed protocol, that collaboration between ants is translated into the use of a cooperative technique between the nodes to mitigate the effects of the channel fading. Cooperative modulation diversity (CMD) exploits diversity gain in a system if each component of the transmitted signal is affected by independent channel fading. Furthermore, to achieve the maximum diversity gain, any two signal points in the system constellation must have the maximum number of distinct components. CMD is invoked in an adaptive manner, since it is needed only if transmission errors occur. The next transmission hop from a source cluster-head can be performed in two stages: broadcast and retransmission.

### 6.1. Broadcast Stage

In the broadcast stage, a source cluster-head forwards its clustered message to another one, *i.e.*, the closest neighbour cluster-head and the next hop in the routing process towards the sink node. The relay candidate, indicated by the relaying threshold, receives the transmitted packet due to the broadcast nature of the wireless channels. Then, the packet is transmitted by a conventional QPSK modulation scheme. The modulated signal is given by [18]

(6) $$s(t)=A\sum_{n=-\infty }^{+\infty }a_{n}p(t-nT_{s})\cos(2\pi f_{c}t)+A\sum_{n=-\infty }^{+\infty }b_{n}p(t-nT_{s})\sin(2\pi f_{c}t)$$

in which

(7) $$\begin{array}{l} a_{n},b_{n}=\;\pm 1\;\text{with equal probability}\\ \;p(t)=\;\{\begin{array}{cc} 1, & 0\leq t\leq T_{s}\\ 0, & \text{otherwise} \end{array} \end{array}$$

for a carrier frequency, *f_c_*, and a carrier amplitude, *A*. The transmitted packet has a Cyclic Redundancy Check (CRC) attached and the receiver detects it. If the packet is correctly detected by the receiver, a positive acknowledgement (ACK) is sent back to the source cluster-head, which remains transmitting new packets and the previous process is repeated. Otherwise, a negative acknowledgement (NACK) is sent back to the source cluster-head and the retransmission stage begins.

### 6.2. Retransmission Stage

The Vice-CH, jointly with the source cluster-head, retransmits the packet using the cooperative modulation diversity. The retransmissions continue until the packet is successfully delivered, or the number of retransmissions exceeds $N_{r}^{\max}$, which is a preset parameter that indicates the maximum number of retransmissions allowed per packet. The value of $N_{r}^{\max}$ may depend on the application [35].

In CMD, if a QPSK constellation is rotated by a certain angle, a kind of redundancy between the two quadrature channels is introduced and the system can take advantage of the resulting diversity. Then, both the source and vice cluster-heads rotate the constellation by an angle *θ*

(8) $$s(t)=A\sum_{n=-\infty }^{+\infty }x_{n}p(t-nT_{s})\cos(2\pi f_{c}t),+A\sum_{n=-\infty }^{+\infty }y_{n}p(t-nT_{s})\sin(2\pi f_{c}t)$$

in which

$$\begin{array}{l} x_{n}=a_{n}\cos\theta -b_{n}\sin\theta \\ y_{n}=b_{n}\sin\theta +b_{n}\cos\theta \end{array}$$

The constant phase *θ* is selected in such a way that the squared Euclidean distance between QPSK signal constellations is maximized for both components, in-phase and quadrature [18].

Quadrature components are generated and each component is independently interleaved. The signal interleavers are chosen such that the two components will be independent after deinterleaving. An example of the interleaving process is presented in Table 2. For simplicity, the interleaving of just two symbols is presented. The first symbol transmitted by the source cluster-head has the quadrature component of the second symbol (*Q*_2_), at the first time slot. Concomitantly, the Vice-CH performs the primary spectrum sensing. At the second time slot, the Vice-CH transmits a symbol with the quadrature component of the first symbol (*Q*_1_), and the source cluster-head performs spectrum sensing. That mechanism enables the detection of a request for emergency reporting, which interrupts the diversity cooperation for (conventionally) transmitting the fused packet and relays the higher priority information.

In that case, the relaying to the next cluster may be performed by the Vice-CH, if the request for emergency reporting arises from a different cluster. Then, the source cluster-head keeps transmitting the rest of the fused packet by simply QPSK. Otherwise, if the request for emergency reporting arises from the same cluster, then the source cluster-head is responsible to relay the correspondent data. As a consequence, the Vice-CH continues the transmission of the fused packet, by simply QPSK.

If no requests for emergency reporting are detected by the spectrum sensing, then both source cluster-head and Vice-CH keep collaborating over CMD. The two components are then upconverted to the carrier frequency and added, using the following expression

(9) $$s_{s}(t)=A\sum_{n=-\infty }^{+\infty }x_{n}p(t-nT_{s})\cos(2\pi f_{c}t)+A\sum_{n=-\infty }^{+\infty }y_{n-k}p(t-nT_{s})\sin(2\pi f_{c}t)$$

in which *k* is an integer representing the time delay in number of symbols introduced by the interleaving between the in-phase and quadrature components.

### 6.3. Channel Model and Decoding System

Consider a communication channel with frequency nonselective slowly fading with a multiplicative factor representing the effect of fading and an additive term representing the Gaussian noise. The received signal is

(10) r(t)=α(t)s(t)+n(t)

in which *α*(*t*) is modelled as zero-mean complex Gaussian process. The received signal, *r*(*t*), is first downconverted to baseband. The obtained signal (lowpass equivalent) in one signalling interval is

(11) $$r_{l}(t)=\alpha_{n}e^{-j\phi_{n}}s_{l}(t)+z(t),\;nT_{s}\leq t\leq (n+1)T_{s}$$

in which *z*(*t*) represents the complex white Gaussian noise, *α_n_* is the fading amplitude (considered constant over one symbol interval), *ϕ*_n_ is the phase shift due to the channel fading, and *s_l_*(*t*) corresponds to the equivalent low pass of the transmitted signal *s*(*t*) [18]. With the phase shift estimation of the received signal at the sink node, and after the demodulation, the received vector is given by

(12) $${\tilde{\text{r}}}_{n}=\alpha_{n}{\text{s}}_{n}+{\text{z}}_{n}$$

in which **s***_n_* is the vector representation of the transmitted signal at time *nT_s_*, and the elements of the complex vector **z***_n_* are independent identically distributed Gaussian random variables with zero mean and variance *N*_0_/2.

The decoded vector at the sink node, after the deinterleaving process, is

(13) rn=αnxn+Re{zn}+j[αn+kyn+Im{zn}]

which is then processed using symbol-by-symbol detection. The optimum demodulator computes the squared Euclidean distance between the received vector and each of the four signal vectors of the QPSK scheme and then decides in favor of the one closest to **r***_n_* [18].

## 7. Performance Evaluation

To evaluate the performance of the proposed techniques, simulations in Matlab 7 are accomplished. The sensor network is composed by 100 nodes, which are distributed over five clusters. Therefore, five cluster-heads and vice cluster-heads are elected in each round. The nodes are deployed randomly on an area of 100 × 100 meters. The sink node is located at the coordinates *x* = 50 and *y* = 250 meters. The bit rate adopted is 128 kbit/s and the length of the packet generated in each sensor node is 128 bits.

**Figure 2.**
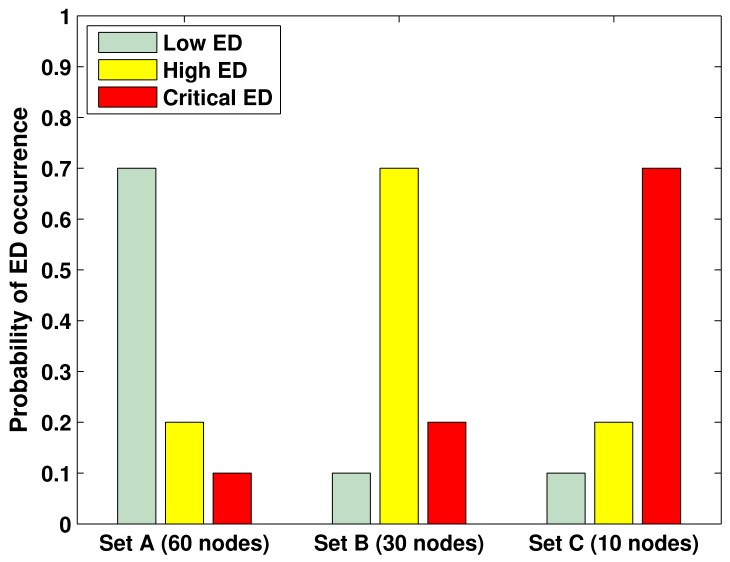
Probability distribution of a node to present a specific emergency degree.

The probability of a node to present a specific emergency degree (*P*_ED_) is distributed in accordance with three different sets. The first set is denoted Set A and contains 60 nodes randomly deployed over the network. The second and third sets are denoted Set B and Set C, and contain 30 and 10 nodes, respectively. The values of *P*_ED_ for the three sets are presented in Figure 2.

The weighting factor, *ω*, is equal to 0.5, because it assures a good balance between the pheromone level and the heuristic information. The heuristic information is relevant as the pheromone level, because it can detect the best positioned nodes. Moreover, *η_s_* has strong influence at the former cluster elections, since *τ_s_* is still similar to all the nodes. Actually, each node only calculates *p_s_κ* after the first round, in which the clusters' elections are performed randomly. After comparing the values of the different decision rules, the ant corresponding to a specific cluster identifies the past path (elects the CH). This is the stop criterion for each cluster.

The fuzzy heuristic information is calculated by the Fuzzy Logic Toolbox of Matlab, and the configured parameters are listed as follows [36]:

- Fuzzy inference system: *Mandani*;
- And method: *min* (minimum);
- Implication: *min* (minimum);
- Aggregation: *max* (maximum);
- Defuzzification: *Centroid*.

The values used to represent the input variables are achieved by a normalization operation, in which the denominator is the circular area around the sensor node. In the simulations, the area radius is 30 m for the variable local_distance and 50 m for the variable CH_dispersion.

Simulation results are presented for average delay time and packet loss rate. The angle of the constellation rotation is selected as *θ* = 27°, because it is optimum for the QPSK scheme [37]. A truncated ARQ is used and the maximum number of retransmissions is four, *i.e.*, $N_{r}^{\max}=4$. A CRC with *C* =16 bits is assumed with a cyclic generator polynomial of G_CRC16_ (*D*) = *D*^16^ + *D*^12^ + *D*^5^ + 1.

It is assumed in this work that the information about the spectrum occupancy and the requests for emergency reporting are available to the sensor nodes from a database search engine [38]. Spectrum sensing data provided to database system can be obtained by classical methods, such as energy detection, cyclostationary feature detection or matched filter detection [39].

Each node has an initial energy of 3 mJ. The radio dissipates *∊*_elec_ = 50 nJ/bit to run the transmitter or receiver circuitry and *∊*_fs_ = 10 pJ/bit/m^2^, or *∊*_mp_ = 0.0013 pJ/bit/m^4^ for the transmitting amplifier to achieve an acceptable $\frac{E_{b}}{N_{0}}$. Consider *d*_0_ as a specific threshold distance, given by

(14) $$d_{0}=\sqrt{\frac{{∊}_{\text{fs}}}{{∊}_{\text{mp}}}}$$

Thus, to transmit a *l*-bit message at a distance *d* using the radio model, the radio spends [40]

(15) $$E_{Tx}(l,d)=\{\begin{array}{cc} l\cdot ({∊}_{\text{elec}}+{∊}_{\text{fs}}\cdot d^{2}), & \text{if}\;d\leq d_{0}\\ l\cdot ({∊}_{\text{elec}}+{∊}_{\text{mp}}\cdot d^{4}), & \text{if}\;d>d_{0} \end{array}$$

and to receive this message, the radio spends

(16) $$E_{Rx}(l)={∊}_{\text{elec}}\cdot l$$

**Figure 3.**
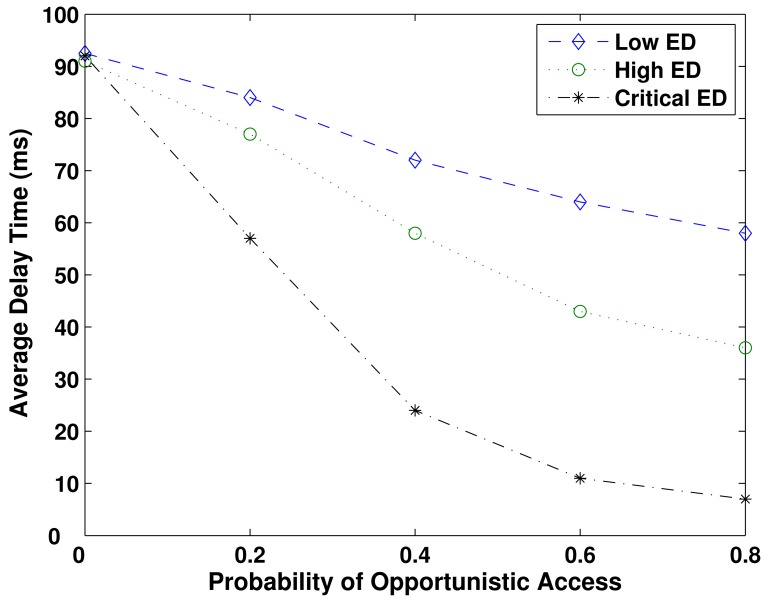
Average delay time as a function of the probability of opportunistic access, over BSR = 0.4. Critical ED nodes present the lowest average delay because they have priority on the resource's usage.

The average delay time (ms) related to the probability of opportunistic access (*P_oa_*), considering the three emergency degrees is evaluated in Figure 3. It decreases as the probability of opportunistic access increases. Users migrate from the secondary network usage to the primary one, which alleviates the high concurrence in the unlicensed spectrum, and improves the quality of service by the use of the idle licensed spectrum. The nodes corresponding to Critical ED present the best performance in all values of *P_oa_*. The lowest value of average delay time is 7 ms, at *P_oa_* = 0.8.

The nodes with High ED present an intermediate performance, because they have less priority and smaller allocation in the opportunistic usage of spectrum, *i.e.*, in this simulation the block size ratio is 0.4. The lowest reached value of average delay time is 36 ms, at *P_oa_* = 0.8. The nodes with Low ED, which are restricted to use the resources of the secondary network, present the worst performance, with the smallest average delay time equals to 58 ms, at *P_oa_* = 0.8. The crowded secondary spectrum contributes to that poorer performance. At *P_oa_* = 0 the users share a similar performance, corresponding to a delay of 91 ms (in average). In this case, due to the null probability of opportunistic access, all the network nodes are restricted to use only the secondary network.

**Figure 4.**
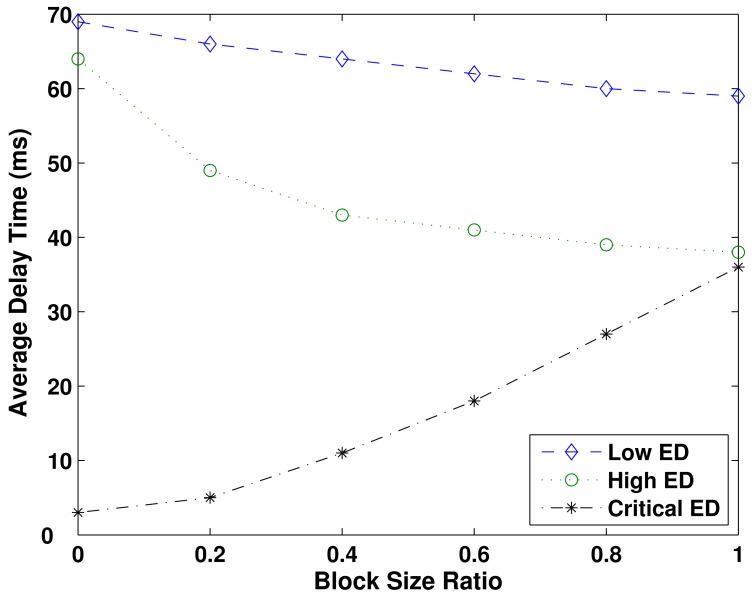
Average delay time as a function of the block size ratio, over *P_oa_* = 0.6. High ED and Critical ED nodes present a similar performance at BSR = 1.

Figure 4 shows the performance evaluation related to the average delay time as a function of the block size ratio, by the assumption of *P_oa_* = 0.6. The nodes with Low ED and High ED have their performance enhanced as the block size ratio increases. That improvement is more expressive in High ED nodes, because they can continuously use the spectrum opportunities for longer time, as the block size ratio increases. If the same block size is assured to Critical ED and to High ED nodes (block size ratio equals to one), then the performance of those nodes is similar (38 ms and 36 ms, respectively). On the other hand, if the block size ratio is 0, then only Critical ED nodes are allowed to use spectrum opportunities in the primary network, which gives the lowest average delay time to those nodes (3 ms). Despite the improvement in the performance of Low ED nodes, the increase in block size ratio does not cause too much impact (compared to the remaining node's classes) in the performance, *i.e.*, only a variation from 69 ms to 59 ms.

**Figure 5.**
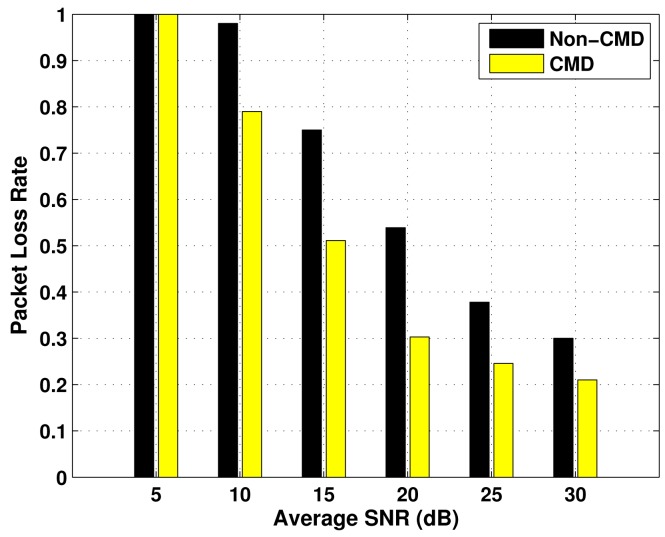
Packet loss rate for Low ED nodes, as a function of the average SNR.

**Figure 6.**
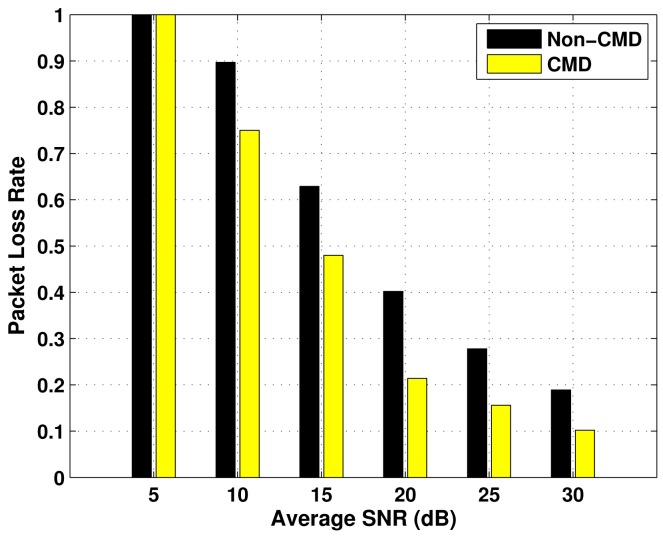
Packet loss rate for High ED nodes, as a function of the average SNR.

**Figure 7.**
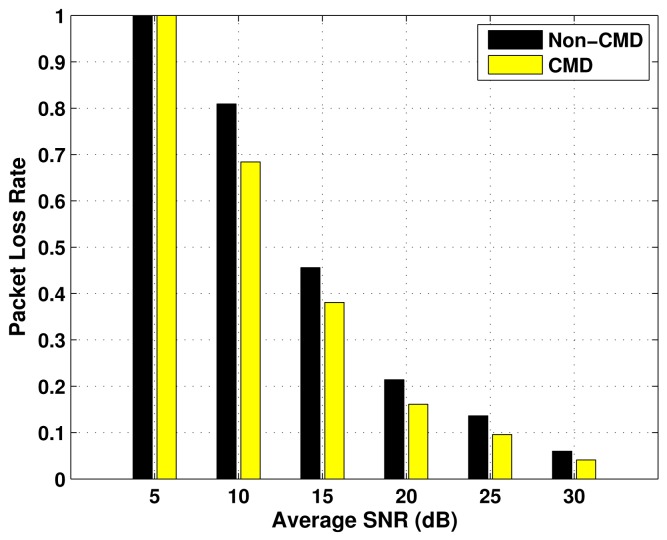
Packet loss rate for Critical ED nodes, as a function of the average SNR.

The results of Packet Loss Rate (PLR) as a function of the average SNR, for the three emergency status considered, are presented in Figures 5–7, over *P_oa_* = 0.6 and BSR = 0.4. In each evaluation, there is a comparison between the performance of the transmission with cooperative modulation diversity (if required), and without diversity (Non-CMD). In all the cases, the values of PLR decrease as the average SNR increases, which is an expected behaviour. Moreover, the higher the emergency degree, the lower the packet loss rate. This occurs because the number of packet collisions is reduced by using vacant spectrum bands according to constraints imposed by the block size ratio.

The use of the cooperative modulation diversity improves the performance of packet loss rate in the three cases of ED. However, the difference between the performance with and without CMD is less expressive in the Critical ED case, because the primary links used by the respective nodes have higher access availability, which leads a small number of collisions. As a consequence, they have better quality of service.

## 8. Conclusions

Healthcare wireless sensor networks present several capabilities for efficient monitoring of patients and for performing emergency reporting. However, they are subjected to high concurrency for free-spectrum access, which decreases the quality of service and damages the emergency reporting to a medical central station. Cognitive radio sensor networks utilize the spectrum more efficiently and they can improve the performance of healthcare networks.

In this paper, the authors introduced the Cognitive LF-Ant protocol, which is inspired by the behaviour of the ants and, by means of a cognitive capability, elects the nodes most suitable to coordinate the operations of a cluster. The cluster-head and the vice cluster-head collaborate to assure the dynamic allocation of resources, in a manner that the patients with higher emergency degrees are privileged. The dynamic resource allocation is inspired by experiments with legs' manipulation performed with desert ants. Moreover, the cooperative modulation diversity with spectrum sensing was proposed to act in an adaptive manner and helps in fading mitigation.

The performance evaluation used simulations in Matlab 7. The results show a decrease in the average delay time required to report emergency situations. Such reduction is related to the increase of the emergency degree. Moreover, the packet loss rate was also reduced, mainly when the cooperative modulation diversity was used.

Future research includes the application of Sugeno inference system, which is also computationally efficient and may work better with optimization and adaptive techniques. Moreover, dynamic generation of fuzzy rules will be designed to deal with the possible changes in the reasoning environment. Furthermore, futures works also include the study of network connectivity, as well as to evaluate the impact of estimation errors in the performance of the proposed protocol.

## Figures and Tables

**Table 1.** Fuzzy rules used in the proposed protocol.

**Rule**	**IF**		**THEN**

	**local_distance**	**CH_dispersion**	**eta**
1	Close	Far	Very High
2	Medium	Far	High
3	Far	Far	Rather High
4	Close	Medium	High Medium
5	Medium	Medium	Medium
6	Far	Medium	Low Medium
7	Close	Close	Rather Low
8	Medium	Close	Low
9	Far	Close	Very Low

**Table 2.** The cooperative interleaving process for two symbols. The spectrum sensing is also used to detect the request for emergency reporting.

	**Time slot 1**		**Time slot 2**	
**Source Cluster-head**	*Q*_2_	*I*_1_	**SS**	
**Vice Cluster-head**	**SS**		*Q*_1_	*I*_2_
